# A Comprehensive Review on the Interaction of Milk Protein Concentrates with Plant-Based Polyphenolics

**DOI:** 10.3390/ijms222413548

**Published:** 2021-12-17

**Authors:** Mansuri M. Tosif, Agnieszka Najda, Aarti Bains, Thummalacharla Chaitanya Krishna, Prince Chawla, Magdalena Dyduch-Siemińska, Joanna Klepacka, Ravinder Kaushik

**Affiliations:** 1 Department of Food Technology and Nutrition, Lovely Professional University, Phagwara 144411, India; tosifmansuri444@gmail.com (M.M.T.); chaitanyakrish1998@gmail.com (T.C.K.); 2 Department of Vegetable and Herbal Crops, University of Life Science in Lublin, Doświadczalna Street 51A, 20-280 Lublin, Poland; 3 Department of Biotechnology, CT Institute of Pharmaceutical Sciences, South Campus, Jalandhar 144020, India; aarti05888@gmail.com; 4 Faculty of Agrobioengineering, Institute of Plant Genetics, Breeding and Biotechnology, University of Life Sciences in Lublin, 15 Akademicka Street, 20-950 Lublin, Poland; magdalena.dyduch@up.lublin.pl; 5 Department of Commodity Science and Food Analysis, Faculty of Food Science, University of Warmia and Mazury in Olsztyn, Oczapowskiego 2, 10-719 Olsztyn, Poland; klepak@uwm.edu.pl; 6 School of Health Sciences, University of Petroleum and Energy Studies, Dehradun 248007, India; ravinder_foodtech2007@rediffmail.com

**Keywords:** (poly)phenols, milk proteins, sodium caseinate, molecular interaction

## Abstract

Functional properties and biological activities of plant-derived polyphenolic compounds have gained great interest due to their epidemiologically proven health benefits and diverse industrial applications in the food and pharmaceutical industry. Moreover, the food processing conditions and certain chemical reactions such as pigmentation, acylation, hydroxylation, and glycosylation can also cause alteration in the stability, antioxidant activity, and structural characteristics of the polyphenolic compounds. Since the (poly)phenols are highly reactive, to overcome these problems, the formulation of a complex of polyphenolic compounds with natural biopolymers is an effective approach. Besides, to increase the bioavailability and bioaccessibility of polyphenolic compounds, milk proteins such as whey protein concentrate, sodium caseinate, and milk protein concentrate act as natural vehicles, due to their specific structural and functional properties with high nutritional value. Therefore, milk proteins are suitable for the delivery of polyphenols to parts of the gastrointestinal tract. Therefore, this review reports on types of (poly)phenols, methods for the analysis of binding interactions between (poly)phenols–milk proteins, and structural changes that occur during the interaction.

## 1. Introduction

(Poly)phenolic compounds are phytochemicals that occur naturally within plant cells, and these components have the potential to improve human health [1]. It has been well studied that bioactive components include several compounds such as flavonoids, alkaloids, antibiotics, and phenolic acids, which can be obtained from different parts of the plants [2]. (poly)phenolics play a major key role in the human diet due to their remarkable therapeutical and excellent biological properties. Furthermore, over the past years, people have been moving towards natural food ingredients, and thus, researchers explored the potential health benefits of plant-derived extracts, and these extracts are often utilized as a vital ingredient in food and nutraceutical products. From a food science perspective, (poly)phenols are vital components that are responsible for the organoleptic and antioxidant properties of food and influence the overall quality attributes of the food [3]. Moreover, (poly)phenols frequently generate a shielding effect to protect the plants from environmental and physiological factors [4,5]. Despite the easy accessibility, availability, and low toxicity of (poly)phenolic compounds, low bioavailability, and rapid metabolism limit the effective utilization of (poly)phenolic compounds [6]. Environmental factors including sunlight, temperature, pH, and oxygen can cause epimerization of bioactive components, which directly limits the utilization of (poly)phenolic components in food materials [7]. Furthermore, the food processing conditions and certain chemical reactions such as pigmentation, acylation, hydroxylation, and glycosylation can also cause an alteration in the stability, antioxidant activity, and structural characteristics of the (poly)phenolic compounds [8]. Since the (poly)phenols are highly reactive, to overcome these problems, the formulation of a complex of (poly)phenolic compounds with natural biopolymers is an effective approach [7]. Over the past years, scientists have reported the enhanced bioactivity and functionalization of (poly)phenolic complexes and among all-natural biopolymers, milk proteins are excessively studied and effectively used for the formulation of a stable complex with several (poly)phenolic compounds. The interactions of (poly)phenols with milk proteins such as sodium caseinate, milk protein concentrate, and whey protein concentrate primarily show hydrogen bonding, hydrophobic interactions, and covalent bonds with other molecules [9]. However, the interactions between milk proteins and another molecule significantly show a great impact on the structure and concentration of (poly)phenols and proteins with alterations in pH, temperature, and ionic strength [10]. The structural and functional properties of the milk proteins may change due to the interaction of (poly)phenols with milk proteins. Therefore, for the formulation of the milk protein-(poly)phenols composites, the mechanism of the interactions of (poly)phenols with milk proteins is important [11]. Moreover, milk proteins effectively help to release the appropriate health-relevant dose of (poly)phenols to the gastrointestinal tract and due to this process, the bioavailability and bio-accessibility of the (poly)phenolic compound also increase [12]. Therefore, in this review, we discussed different detrimental effects on plant-based (poly)phenolic compounds. Additionally, the possible mechanisms of the interaction of (poly)phenolic components with milk protein are discussed. The effect of interaction on the functionality and stability of (poly)phenols and structural changes of milk protein is elaboratively discussed. The application of the formulated complex and methods for the analysis of binding interactions between (poly)phenols–milk proteins such as ultrafiltration, isothermal titration calorimetry, molecular docking is also discussed in detail.

## 2. (Poly)phenolic Compounds

**Figure 1:**
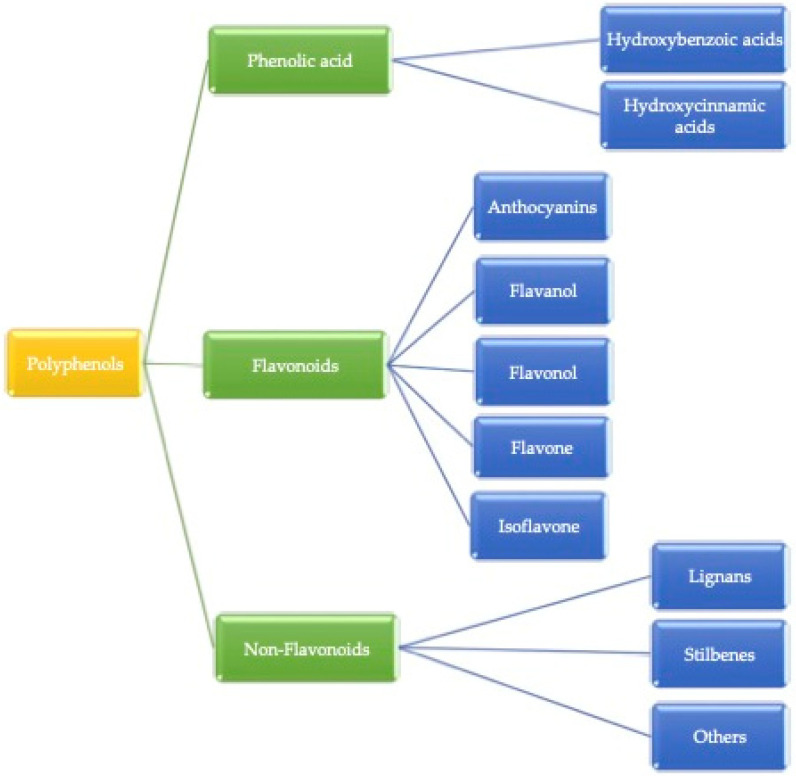
Classification of (poly)phenols.

Plant-based food contains many important micro and macro components for the growth, development, and health of human beings [13]. These compounds are heterogeneous in nature and consist of phenolic acids, stilbenes, lignans, and flavonoids [14]. Factors such as the release of bioactive compounds from plant material, solubility, passage across intestinal epithelial cells, as well as chemical and enzymatic reactions occurring in the gastrointestinal tract are affected by bioavailability [15]. The secondary metabolites, bioactive compounds, are a good source of nutritional value with interesting biological activities in plant-based foods and mainly occur in the presence of (poly)phenols. Alkaloids, flavonoids, stilbenes, lignans, phenolic acids, and others are different kinds of bioactive compounds present in plants. The major group of secondary metabolites we found is mostly (poly)phenolic compounds. Some of the plant-based foods in which (poly)phenol compounds can be found are fruits, vegetables, coffee, tea, wine, cereal grains, and berries [16]. (Poly)phenols are categorized into three parts, as shown in Figure 1. Gallic acid is the most common example of hydroxybenzoic acid, and ferulic acid is considered a hydroxycinnamic acid. Additionally, Phenolic acids are simple phenols that contain a carboxyl group and occur mainly as hydroxybenzoic (C6-C1 skeleton) and hydroxycinnamic acids (C6-C3 skeleton), which derive from benzoic or cinnamic acid, respectively [17]. Herein, hydroxycinnamic acid compounds occur most frequently as simple esters with hydroxy carboxylic acids or glucose, while the hydroxybenzoic acid compounds are present mainly in the form of glucosides, whereas chemically flavonoids are based on a fifteen-carbon skeleton consisting of two benzene rings linked via a heterocyclic pyrene ring [9]. However, non-flavonoids mainly consist of stilbenes, phenolic acids, and tannins, and tannins can be further divided into gallotannin, ellagitannin, hydrolyzed and condensed tannin, and so on. The basic structural difference between these classes is that non-flavonoids contain only one phenol ring and flavonoids contain two phenol rings [18].

### 2.1. Types of (Poly)phenols

These (poly)phenols are divided into different groups based on the function of the phenolic rings, which present these structural elements and bind the rings to each other. Generally, (poly)phenolic compounds are classified into three major groups: (I) flavonoids (II) phenolic acids, and (III) non-flavonoids.

#### 2.1.1. Flavonoids

**Figure 2:**
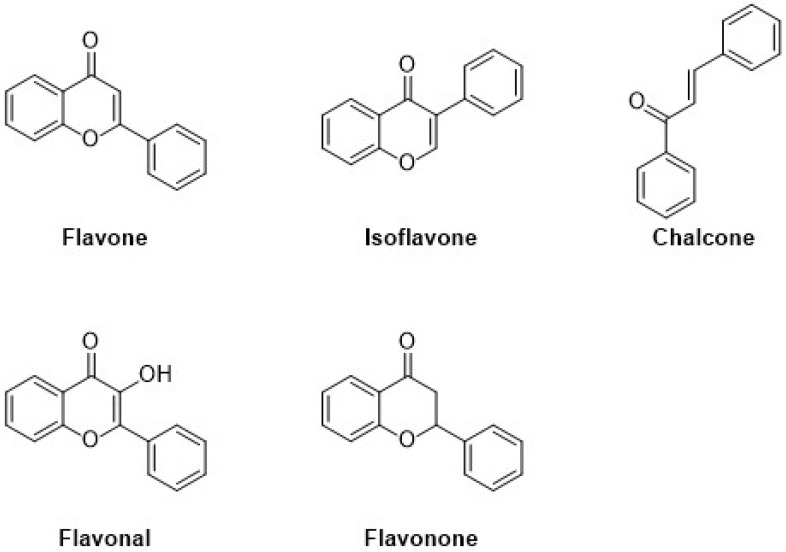
Classification and molecular structures with the examples of common plant-derived flavonoids.

Flavonoids are the major group of (poly)phenolic compounds that occur naturally. Based on their chemical components, they are present in six different subgroups, i.e., flavanols, flavones, isoflavones, anthocyanin, flavanones, and chalcone. Flavonoids contain a three-ring structure with multiple replacements, and thus, due to their structural composition, their molecular weight is very low, as shown in Figure 2. [19]. An extensive range of pharmacological functions in addition to antioxidant, antibacterial, hepatoprotective, and anti-inflammatory effects are present in flavonoids [20]. These flavonoids represent a basic structure of two phenyl groups linked with three-carbon atoms, which generally form with oxygen and are linked with glycosidic linkage. In many conditions, there are three or more hydroxyl groups linked to the structure of the backbone [21].

#### 2.1.2. Phenolic Acids

**Figure 3:**
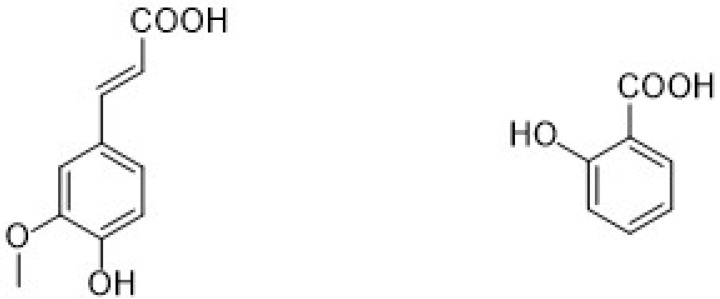
Chemical structure of different types of phenolic acids (hydroxycinnamic acids are hydroxy metabolites with a C6-C3 backbone, and hydroxybenzoic acid is a monohydroxy benzoic acid carrying a hydroxy substituent at C4 of the benzene ring).

**Figure 4:**
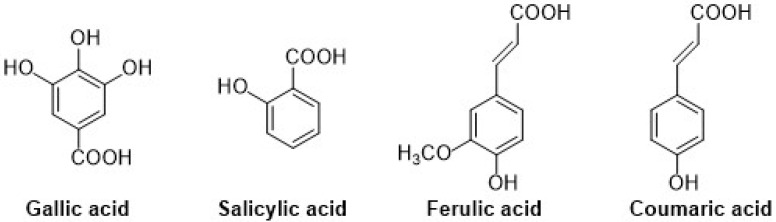
Chemical structure of gallic (hydroxy group at 3rd, 4th, and 5th position), salicylic (hydroxy group at ortho position), ferulic (hydroxy group at 3rd and 4th position on phenyl ring), and coumaric acids (hydroxy group at 4th position on phenyl ring).

Different types of phenolic acids are commonly available in fruits and vegetables, whereas bound phenolic acids are present in grains and their derivatives [22]. The phenolic acids can be divided into two groups: cinnamic acid and benzoic acids. Cinnamic acid consists of nine (C6-C3) carbon atoms which are also known as phenylpropanoids [23], and benzoic acid contains seven (C6-C1) carbon atoms. Moreover, they exist mostly as hydroxycinnamic and hydroxybenzoic acids, which are available either in conjugated or free forms, as shown in Figure 3. In addition, hydroxycinnamic acids such as P-coumaric acid (C_9_H_8_O_3_), ferulic acid (C_10_H_10_O_4_), sinapic acid (C_11_H_12_O_5_), gallic acid (C_7_H_6_O_5_), and salicylic acid (C_7_H_6_O_3_) are also abundantly available in different types of foods (Figure 4). These acids also show radical scavenging activity to stabilize the resulting phenoxyl radicals within their structure by donating electrons. [24]. Furthermore, hydroxybenzoic acid (4-HBA) can be utilized to formulate value-added bioproducts that have great potential application in the pharmacy, food, and agricultural pesticide industries. Besides, ellagic acid, mostly available in cranberries, blueberries, strawberries, and blackberries, shows potential effects that decrease high blood cholesterol, maintain blood pressure, and smooth skin wrinkles from radiation. Gallic acid is mostly known for its antioxidant effect and is available in mango, soy, and tea [25].

#### 2.1.3. Non-Flavonoids

Tannins, stilbenes, xanthones, and lignans are considered as the non-flavonoid phenolic group. Most of these phenolic compounds contain at least two aromatic rings in their structure, whereas tannins contain more than two aromatic rings in their structure. However, lignans are vascular plant’s secondary metabolites; with a wide range of functional properties, these are derived from the combination of two cinnamic acid units of C6-C3 in β-carbon atoms, which are linked with an additional lactone or ether bond [19]. Stilbenes are also non-flavonoid compounds that are present in almonds, beans, blueberries, peanuts, and wine, respectively [26].

### 2.2. Extraction Process

Currently, several techniques including the solvent extraction method, liquid-liquid extraction method (LLE), pressurized liquid extraction method (PLE), ultrasonic-assisted extraction method (UAE), microwave-assisted extraction method (MAE), and supercritical fluid extraction method (SFE) have been exploited to extract secondary metabolites (polyphenols) from various sources [27]. However, liquid-liquid extraction and solid-liquid extraction techniques are effectively used for the extraction of (poly)phenols. Moreover, various factors such as chemical structure, time duration, type of solvent, and the polarity of compounds always play a key role in the extraction of (poly)phenolics. Several studies also suggested that stabilizers can be used during the extraction of (poly)phenols to retain effective functional properties [28]. Some suitable solvents, such as alcohol, methanol, and ethanol, are used for the extraction of (poly)phenols from natural sources [12]. Furthermore, the purity of (poly)phenols, yield, and variation in rate is highly dependent upon the extraction techniques. Although solvent-based extraction produces larger yields, it restricts the use of (poly)phenols for human consumption. (Poly)phenols must be purified further after solvent extraction, either through the membrane or ultrafiltration. Thus, to avoid degradation, the solvent extraction method was devised to separate soluble chemicals from a solid matrix (plant tissue) using a liquid matrix (solvent) at a lower temperature. The choice of solvent, together with heating and/or agitation, is critical for the solvent extraction of bioactive chemicals and antioxidants from plant materials [24]. The use of different solvents leads to a change in the composition of the extract. Besides, the effects of extraction time and temperature also influence the total (poly)phenol content and antioxidant activity of tea leaves [29].

### 2.3. Factors Causing Detrimental Effects on the Bioactivity of (Poly)phenols

(Poly)phenolic extracts are “generally recognized as safe (GRAS),” and (poly)phenol can impact biological entities such as cells, organs, and organisms. The health effects of (poly)phenols ultimately depend upon dose intake according to the age of the consumer [30]. Commercially, several products rich in (poly)phenols are manufactured by many industries; and the recommended isoflavones consumption rate is 50 mg/day, and the consumption of grape seed extract rich in proanthocyanins is 100–300 mg/day. Additionally, some authors have stated several aspects about the intake of higher concentrations of (poly)phenolic compounds that show the impact on the different biochemical processes, leading to side effects [30]. Excessive (poly)phenolic compound consumption is linked with nephrotoxicity and hepatotoxicity, and they can cause mutation to cells that may cause cancer or negatively impact the regulation of the thyroid hormone [31]. The consumption of (poly)phenolic compounds inhibits the engagement of nonheme iron, resulting in the reduction in microelements in populations at risk due to higher consumption of tea. Quercetin enhances the redox cycling of oestradiol-induced tumorigenesis and catechol estrogens.

#### 2.3.1. Effect of Temperature on (Poly)phenols

The temperature has a great influence on the processing and storage of (poly)phenolic-rich food products, and these (poly)phenolic compounds may undergo an epimerization process. To control this epimerization, the appropriate temperature is critical to maintaining the (poly)phenol levels as they were diminished by 20–21% after undergoing heating at 70 °C for 30 min [32]. To this point, the majority of epigallocatechin gallate and some associated catechins in green tea leaves are epimerized during the brewing process. The epimerization process reverses the stereochemistry of the bond that bridges the B- and C-rings and converts epigallocatechin gallate (EGCG) to gallocatechin gallate (GCG). Accordingly, a significant amount of epigallocatechin gallate consumed during the consumption of green tea is gallocatechin gallate. In another study, catechin was diminished up to 20% when it was brewed for 7 h at 98 °C, and it was stable at room temperature [17]. Moreover, some experimental results suggest that (poly)phenols can directly be affected by storage conditions, mainly due to oxidation, hydrolysis, and complexations. However, the (poly)phenol compound maintains a stable structure for a longer time, and it also suppresses the (poly)phenol oxidase enzyme activity at low temperatures (4 °C) [33].

#### 2.3.2. Effect of pH on (Poly)phenols

**Figure 5:**
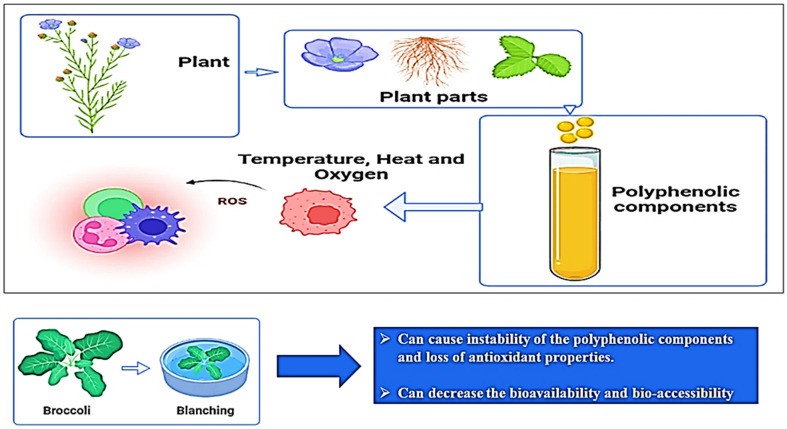
Effect of temperature on the stability of plant-based (poly)phenolic components.

pH is another factor that influences (poly)phenol stability in fruits and vegetables that experience changes in the chemical form of (poly)phenols, which is also a reason for color variation in (poly)phenols. In general, th (poly)phenols are more stable at a low pH, which also reflects on the absorption of (poly)phenols in the human digestive system. For example, the extract of millet seed coat remains stable at a pH of 6.5, and they are unstable at a pH of 10. Moreover, green tea catechins are stable when the pH is below 4 and unstable at a pH greater than 6 in an aqueous solution. The effects of temperature and pH on (poly)phenols are shown in Figure 5 [33].

#### 2.3.3. Effect of Oxygen on (Poly)phenols

**Figure 6:**
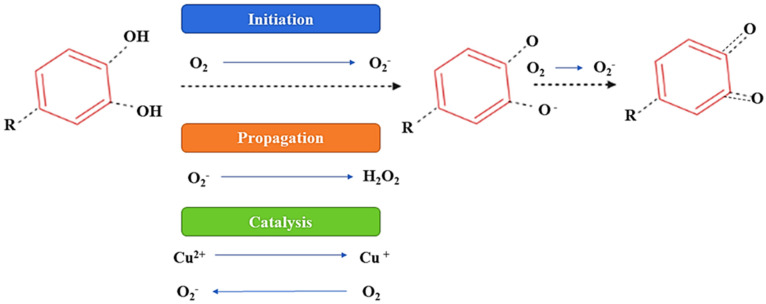
Proposed mechanism of autoxidation of (poly)phenolic components.

In the presence of oxygen, (poly)phenolic contents can attain autooxidation, which is the major factor of (poly)phenols’ instability, and as a result of autooxidation, hydroperoxides and peroxides are formed. Moreover, autoxidation leads to oxidative degradation and a significant decline in the concentration and bioactivity of (poly)phenols [33,34]. This activity of a (poly)phenolic compound is primarily associated with the number of OH groups, molecular structures, the resonance effects, and the double-bond conjugation [34]. The imbalance between oxidative and reductive processes is caused by enzymatic browning due to the presence of oxygen, and to overcome this problem the application of vacuum conditions or a modified atmosphere (N_2_ or CO_2_) is recommended [35]. Furthermore, it has been also reported that (poly)phenolic components promote oxidative damage to DNA, lipids, and deoxyribose in the presence of bivalent metallic components. These effects are suspected to result from the autoxidation process and have also been suggested to induce mutagenesis, carcinogenesis, or the promotion of cancer [33,34,35]. Furthermore, the consumption of food rich in (poly)phenolic compounds leads to the generation of substantial amounts of H_2_O_2_ [34,35]. Figure 6 proposes a possible mechanism of the autoxidation of the (poly)phenolic compounds.

#### 2.3.4. Effect of Light on (Poly)phenols

Another reason for (poly)phenol degradation is the influence of light; uncontrolled light can cause (poly)phenol isomerization, i.e., the effects of bathochromic and hyperchromic shifts can cause the degradation of (poly)phenols by UV light [36]. The light affects the changes in the structure of (poly)phenols, and to avoid this degradation the extracted (poly)phenols are stored under dark conditions.

## 3. Milk Proteins

Protein-(poly)phenol interactions are linked to protein surface properties, and protein conformation is just as crucial as (poly)phenol structure in determining the type of the binding reaction. However, a broad selection of existing ingredients such as carbohydrates, fats, and proteins is being promoted for several industrial applications. Additionally, milk proteins have a higher nutritional value and also exceptional physicochemical properties and due to this, they are used as primary functional components in many foods. Additionally, several forms of milk proteins are commercially available such as caseins, caseinates, milk protein concentrates (MPCs), whey protein isolates (WPIs), and whey protein concentrate (WPCs), which are produced from milk products. MPCs are directly prepared using diafiltration or ultrafiltration methods on skim milk [37]. Milk protein that precipitates the solution under the isoelectric pH 4.6 at 30 °C is known as casein. In milk proteins, caseins have an amorphous structure and high proline content. A(s1)-casein, α(s2)-casein, β-casein, and kappa-casein are prime fractions, and hydrophobic interactions, hydrogen bonding, and calcium phosphate bridge them together to form casein micelles [38]. Moreover, caseins are used as carriers for the direct delivery of several bioactive compounds, and their physicochemical and structural properties enable them to serve in body delivery systems.

In addition, casein micelles contain many hydrophobic- and hydrophilic-bound bioactive compounds and metallic ions. It also exerts excellent self-assembling, surface properties, and gelation properties that make the interaction of other macromolecules form a complex structure with distinctive properties, which are important for protecting sensitive payload [9,10,11]. Moreover, the combination of extracted casein with a sodium molecule resulted in sodium caseinate, which is a multifunctional milk protein. In comparison with other types of caseinates, sodium caseinate has a higher protein content and is the most water-soluble [10]. Caseins exhibit good compatibility with other proteins and ligands due to suitable open structures and high hydrophobic areas [37,38]. Whey proteins, also known as serum proteins, are globular proteins that contribute 20% of the total protein in bovine milk and practically all of the protein in whey. They are complex-structured proteins with a high level of secondary and tertiary protein structure [9,10,11]. In addition, the by-products of cheese production are whey proteins, and these proteins are disposed of as waste, which increases environmental and food sustainability issues. To avoid these issues, the utilization of whey proteins from food waste to prepare beneficial products using different separation technologies could be a better approach. From the nutritional perspective, whey protein hydrolysates (WPHs) are well absorbed and digested in the gastrointestinal tract [39]. The addition of (poly)phenols can influence the functional properties of whey proteins. The solubility, foam stability, and foam capability are improved when chlorogenic acid interacts with the whey proteins [40]. The formation of a complex between whey proteins and cinnamon can terminate the production of the tumor necrosis factor (TNF-α) [41].

### 3.1. Interaction of Milk Proteins with (Poly)phenols

**Figure 7:**
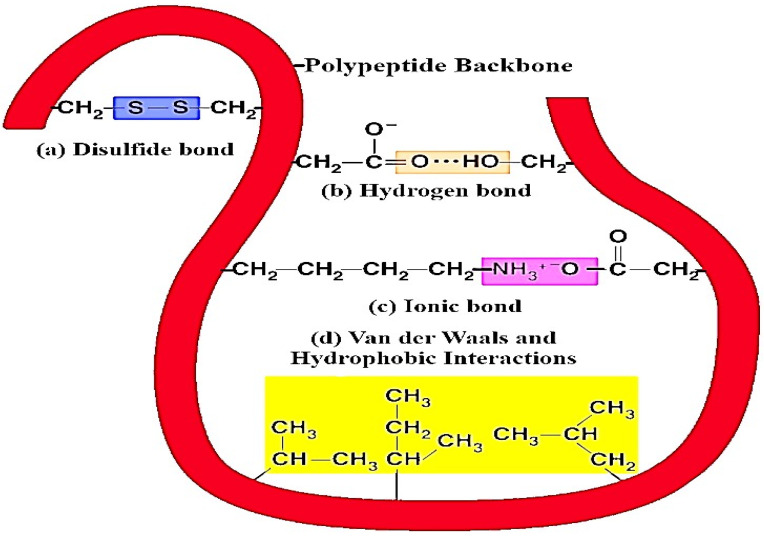
Different types of intermolecular and intramolecular interaction between protein molecules and plant-based (poly)phenolic components. Herein, (a) disulfide bonds function to stabilize the quaternary structure of milk proteins and (poly)phenols, (b) hydrogen bonds provide most of the directional interaction that underpins milk proteins folding with (poly)phenols, and (c) ionic bonds are potent electrostatic attractions; therefore, they form as atoms of amino acids bearing opposite electrical charges, (d) hydrophobic interactions allow the milk protein surface to decrease and also reduce the undesirable interaction between (poly)phenols.

Milk proteins are extensively accessible, low-cost, natural raw materials with high nutritional value and good sensory attributes [42]. These milk proteins can bind with various kinds of molecules with different degrees of affinity due to their hydrophobic sites. The various forces of interaction such as hydrogen bonds, ionic, hydrophobic interactions, and van der Waals forces (as shown in Figure 7 and Table 1) play a significant role in the binding of milk proteins with different types of (poly)phenolic components [4]. Furthermore, in non-covalent binding, the hydrophobic molecules are bounded through hydrogen bonding, and the binding sites are situated on the surface of the proteins. This mechanism is useful for the transport of hydrophobic forms of vitamins, fatty acids, or (poly)phenolic compounds. In this context, Yuksel et al. [43] characterized the binding interactions between milk protein (β-casein) and green tea flavonoids (catechin). In their study, isothermal titration calorimetry and fluorescent probe binding methods were used to evaluate binding interactions between milk proteins and green tea flavonoids. The results of the study showed decreases in the hydrophobic sites’ surfaces due to the presence of green tea flavonoids for all the casein and solid-non-fat concentrations. However, isothermal titration calorimetry confirmed the non-covalent bonding between β-casein and catechin. In covalent bonding, the hydrophobic substances are attached to free reactive functional groups on the surface or inner cavities of milk proteins [44]. Caseins have a strong binding affinity to interact with (poly)phenols and are also convenient for nanoencapsulation [18]. Functional properties such as solubility and the stability of milk proteins may change when they interact with macromolecules. To avoid these issues, succinylation is a good choice for the modification of proteins. By using this method, the milk proteins improve their chemical, functional, and biological properties [45]. Similarly, Kanakis et al. [46] conducted a study for the evaluation of the interaction between β-lactoglobulin and (poly)phenols (catechin, epicatechin, epigallocatechin, and epigallocatechin gallate) by using various techniques including molecular modeling and fluorescence spectroscopic methods CD and FTIR. Moreover, an effect of (poly)phenol complexation, binding constant, and (poly)phenol binding mode on β-lactoglobulin and the secondary structure was determined. Herein, the structural study confirmed the binding of β-lactoglobulin and (poly)phenols via both hydrophobic and hydrophilic interactions.

#### 3.1.1. Non-Covalent Interaction between Milk Proteins and (Poly)phenols

Non-covalent interactions are mostly reversible reactions between proteins and (poly)phenols, and they are weaker than their covalent counterparts [46]. Generally, hydrophobic interactions and hydrogen bonding are principally involved in the interaction between milk proteins and (poly)phenols. The phenolic groups are known as good donors of hydrogen and form hydrogen bonds with carbon-oxygen groups of proteins [47]. Hydrogen bonds may form when the interaction takes place between OH groups of (poly)phenols and O_2_ or N_2_, specifically OH and NH_2_ groups of proteins [38]. The interaction between caseins and catechins can occur through residues of amino acid chains by hydrogen bonds and hydrophobic interactions [48]. Several amino acids such as glycine, valine, tyrosine, isoleucine, leucine, cysteine, phenylalanine, alanine, tryptophan, and methionine residues of proteins are considered hydrophobic amino acids sites that interact with non-polar aromatic rings of (poly)phenolic compounds [49]. The ionic bonds play a small role in the interaction of positively charged lysine and amino acid reacts with negatively charged OH groups of (poly)phenols. The non-covalent interactions between milk proteins and (poly)phenols play a key role in improving the functionality and quality of food products.

#### 3.1.2. Covalent Interaction between Milk Proteins and (Poly)phenol

The covalent interactions between proteins and (poly)phenols usually occur through C-N or C-S bonding. One of the most common non-enzymatic methods used for the conjugation of protein (poly)phenol interaction is known as the alkaline reaction [50]. Under alkaline conditions, (poly)phenols are prone to oxidize at a pH range of 9 in the presence of O_2_. These (poly)phenols are converted into semi-quinone radicals, and then they again change into quinones. These intermediate products can readily react with the amino acid residues in the protein side chains. The reaction between proteins and (poly)phenols can be formed by covalent cross-linkage [51].

## 4. Functional Properties of (Poly)phenol–Milk Protein Complexes

The (poly)phenolic compounds and protein molecules show their functional properties. The formation of milk protein-(poly)phenolic complexes exhibits unique and multi-functional characteristics. These complexes show several functional properties such as gelling, solubility, thermal stability, and changes in the functional properties of the representative protein-(poly)phenolic complexes are concise.

### 4.1. Solubility

The solubility of protein is a significant property for assessing many functionalities of protein molecules as the insoluble protein cannot be used as a suitable ingredient for food product manufacturing. The complex of milk proteins and (poly)phenols could either increase or decrease the water solubility properties of proteins, which is dependent on the nature of the complex formation. Furthermore, interaction can influence the surface charge of the protein molecule, which can also change the isoelectric point, and this variation leads to changes in the pH and solubility properties of the complexes [51]. Furthermore, the interaction of non-polar (poly)phenolic compounds with the proteins increases the surface hydrophobicity of protein molecules by reducing the water solubility property [49]. It has been reported that when the milk proteins interact with chlorogenic acid non-covalently, the protein solubility of whey protein isolate and casein increases [40]. Moreover, at pH ≥ 8, there is a decrease in the solubility of lysozyme in the presence of chlorogenic acid in the milk protein. Therefore, the solubility property of (poly)phenol–protein complexes are highly influenced by the type of protein molecule and the type and structure of (poly)phenols and pH.

### 4.2. Thermal Stability

Thermal stability is another key property in the formation of (poly)phenol–milk proteins complexes. There is an improvement in thermal stability for globular proteins through the preparation of (poly)phenol–milk protein complexes. Enthalpy changes occur during the complex formation between milk proteins and (poly)phenolic compounds. At pH 7.4, the interaction of ferulic acid with bovine serum albumin (BSA) exhibits an increase in the melting temperature of native BSA. This shows that the binding of ferulic acid increases the thermal stability of serum albumin protein [52]. However, the interaction of chlorogenic acid and epigallocatechin gallate with lactoferrin can prevent the thermal assembling of lactoferrin at neutral pH [53].

### 4.3. Gelation

Several plants (poly)phenolic compounds can interact with protein molecules for the formation of complexes and can be converted into a gel-like structure with significantly enhanced properties. When the gels are formed by covalent interactions, they become are firmer and thermally stable. The major protein in whey is β-lactoglobulin, and its primary gelling agent dominates the thermal behavior of the total whey protein system. In addition, β-lactoglobulin modified by green tea (poly)phenols exhibited an increased gelation property when the gelling temperature and gelling time were decreased. The formation of the tea (poly)phenol–whey protein complex affected the rheological properties of the gels [54]. The formation of the (poly)phenol–milk protein complexes under covalent interactions can produce the enhanced gelation properties of the complexes.

## 5. Factors Affecting Binding Interactions between (Poly)phenols and Protein Complexes

The formation of protein-phenolic complexes may have a significant influence on protein structure, solubility, hydrophobicity, thermal stability, and the isoelectric point, and some environmental and food processing conditions such as ionic strength, pH, temperature, and others can affect the interaction between (poly)phenols and milk protein complexes [49]. The type of protein complex and the structure of (poly)phenols are also primary factors that can affect the binding interactions of (poly)phenol–protein complexes [46].

### 5.1. Ionic Strength and pH

The pH-dependent changes in binding affinity and the characteristics of (poly)phenols seem to be directly associated with the structural variations of protein molecules during pH changes, indicating an indirect influence on milk protein-(poly)phenol interactions. Several reports revealed that the interaction of (poly)phenols with milk proteins is affected by the pH. At a low pH level, chlorogenic acids were bound to bovine serum albumin (BSA) without any changes in the binding affinity. So, it was confirmed that there was no effect on binding interactions when the pH value ranged from 3–7. However, a lack of available binding sites for chlorogenic acid and ferulic acid may change the tertiary structures of BSA at a very low pH [10]. Moreover, (poly)phenols exhibit a significant interaction with proteins with an isoelectric point for the interaction [55]. The binding affinity and (poly)phenol attributes that appear to be associated with the conformational changes of proteins undergoing the pH change, show the indirect effect on the interaction of (poly)phenol–proteins [49].

### 5.2. Temperature

Temperature changes have been shown to influence protein phenolic interactions by causing structural changes in protein molecules as well as the solubility of the ligand. The impact of temperature on (poly)phenol–milk protein interaction may vary, which is highly dependent on the structure of the protein and the main driving force of binding [32]. Various heat treatment standards are commonly used in the dairy industry to produce numerous products. Similarly, whey protein denaturation in milk affects the techno-functional characteristics of dairy products. As a result, understanding the effects of temperature and the thermal denaturation of milk proteins on (poly)phenol binding is crucial, particularly for optimizing process conditions when (poly)phenol-rich dairy products are required [9]. The temperature may cause many structural changes in (poly)phenols as well as protein molecules [55]. The increase in temperature may cause the denaturation of bovine serum albumin and due to protein denaturation, the binding efficiency of proteins can be reduced; however, α-lactalbumin did not show changes in the binding sites after denaturation. Furthermore, at high temperatures, the hydrophobic binding sites are available due to the unfolding of the protein, showing the greater binding levels of EGCG on the surface of bovine albumin serum (BSA). During preheating, α-lactalbumin exhibits significant interaction with EGCG when compared to the original state of αlactalbumin. After heat denaturation, the surface area of BSA is decreased. The binding of quercetin and chlorogenic acid with BSA shows weak binding due to the polymerization of the bovine serum albumin protein molecule. Additionally, the denatured α-lactalbumin can bind with chlorogenic acid at a greater affinity due to the availability of the exposed residues of amino acids [10]. Therefore, the temperature effect on the interactions of (poly)phenols with milk proteins may be different and depends upon the structure and binding of the compounds.

### 5.3. Type of Protein Complex

During the interactions of (poly)phenol with protein and for the formation of complexes, the surface property of milk proteins plays a vital role. Naturally, the amorphous nature of proteins indicates they may have a higher affinity than structured proteins or globular proteins; however, the BSA consists of binding sites for (poly)phenolic compounds even with the globular structure. Furthermore, in BSA, the composition of amino acids, especially proline and prolyl residues, plays a prominent role in the binding properties of proteins with phenolic compounds [38]. The existence of the massive orientation of amino acids in a globular position may reduce the binding of phenolic compounds, which leads to the low accessibility of (poly)phenols for the protein molecule. The high proline residues of milk casein proteins are treated as a unique vehicle for phenolic compounds. Due to similar molecular weight, net charges, and size, casein fractions are useful for different functional properties. At lower concentrations, β-caseins can form reversible micelles. Epigallocatechin gallate exhibits a greater affinity for β-caseins than other proteins such as lysozyme, ovalbumin, α-lactalbumin, β-lactoglobulin, and gelatin. The binding of genistein and resveratrol on casein proteins such as α-casein and β-casein showed the variations for each casein fraction, and the stability of the β-casein with flavonoid is higher than that with αcasein [38]. Generally, β-casein shows a higher affinity than α-casein due to the presence of higher proline repeats and proline content in the composition of amino acids and the higher hydrophobic nature of β-casein [56]. Additionally, proteins with higher molecular weight may show a higher affinity for (poly)phenolic compounds.

### 5.4. Structure of (Poly)phenolic Compound

The binding properties and affinities of (poly)phenolic compounds to milk protein for the formation of complexes are affected by its (poly)phenolic nature and its structure. These (poly)phenols differ in hydrophobicity, hydroxylation, glycosylation, methylation, molecular weight, and flexibility, and these properties all play an important role in the formation of (poly)phenol and protein complexes [57]. According to several studies, the binding affinity of phenolic compounds increases with an increase in the molecular weight of (poly)phenols [56]. Compared with the polyglycoside forms of flavonoids, monoglycoside forms exhibit a strong binding affinity for milk proteins. When the process of hydroxylation on the C-ring of flavonoids is increased then it shows higher binding factors with bovine albumin serum (BSA). This shows the importance of hydrogen bonds between flavonoids and BSA polar groups. There is an improvement in the binding of flavonoids due to the presence of the C2-C3 double bond, which helps the flavonoid to interact with buried sites of BSA. Due to the structural diversity of (poly)phenolic compounds and different substitution levels, as well as cis-trans isomerism, one has to consider the main factor of the formation of protein and (poly)phenolic complex studies. However, the structure of both (poly)phenol and protein molecules are key factors affecting the binding interactions.

## 6. Significances of Binding Reactions of Protein and (Poly)phenolic Complexes

The binding of (poly)phenols on proteins may produce several results, with changes in the protein structures and also some of the functional, nutritional, and digestibility properties. While some proteins and the (poly)phenolic covalent interactions between them are rare to observe, the covalent interaction effects are more noticeable when compared to the noncovalent binding.

### Structural Changes

The binding of secondary or tertiary structures of proteins to small molecules can be analyzed by circular dichroism (CD) and Fourier transforms infrared spectroscopy (FTIR) techniques. Many studies discussed the effects of (poly)phenols on the structure of a protein. When bovine serum albumin (BSA) interacts with epicatechin, catechin, epicatechin gallate, and tannic acid, there is no change in the protein structure [58]. β-casein interacts with the quercetin without any protein structure changes. Additionally, the protein structure remains unchanged when β-lactoglobulin binds with naringenin and tannic acid [59]. When β-lactoglobulin binds with epigallocatechin gallate, there is a slight change in the secondary structure of a protein [60]. There is an influence on the structure and noncovalent interactions between BSA and flavonoids by quercetin and rutin, but the secondary structure of BSA remains stable. The interaction between β-lactoglobulin and catechins increases the structural stabilization of the protein with α-helix and β-sheet content. The interaction between the different types of flavonoids and BSA and the binding of epicatechin-3-gallate with protein changes the secondary structure of BSA and increases its α-helix content [61]. The changes in protein structures when they bind with the (poly)phenols are pH-dependent, and the interaction with the (poly)phenolic complexes at a pH range between 2.5 and 7.2; there is no change in the structure of β-lactoglobulin. The protein destabilization was reported with an increase in α-helix at a pH of 1.2 [62]. (Poly)phenolic concentration is an important factor that affects the protein structure after the binding reaction. No changes in the secondary structures of β-lactoglobulin occur when the double concentrations of EGCG react with protein. In the case of acidic pH, the effect of epigallocatechin gallate on a β-lactoglobulin structure is much less abundant. After binding EGCG with β-lactoglobulin, the change in the structure of the protein causes a slight increase in the α-helix structure [63]. The decrease in the β-sheet structure is due to an increase in turn in the structure that may cause the binding of resveratrol and curcumin with the incomplete destabilization of β-lactoglobulin [64]. A slight increase in the β-sheet and decrease in the αhelix structure of caseins are effects of binding α-casein and β-casein with (poly)phenols. There is no change in the structure of α-casein in the binding reaction [65]. The binding of two catechins and epicatechins with bovine serum albumin (BSA) shows a decrease in the β-sheet and an increase in α-helix structure. Significantly, the protein structural changes upon binding with (poly)phenols are not only functional properties but also binding reactions [66]. In noncovalent interactions, the structures of milk protein are modified. These changes are mostly influenced by pH and (poly)phenol concentration, which contrasts with the results of various studies. In noncovalent interactions, the structures of milk protein are modified. These changes are mostly influenced by pH and (poly)phenol concentration, which contrasts with the results of various studies.

## 7. Analysis of Protein and (Poly)phenolic Binding Interactions

The binding of (poly)phenols with milk proteins has a significant effect on covalent and noncovalent interactions. Various technologies help to analyze the interaction of (poly)phenols with milk proteins by using some analytical methods. The overview of binding reactions between (poly)phenols and milk proteins including some methodologies used for the analysis of the binding interactions is given below.

### 7.1. Ultrafiltration

The evaluation of the protein-ligand binding uses direct and indirect methods that have been widely used since the 1970s. The direct method is used for measuring the complexes, and the indirect method is used for measuring the thermodynamic activity that occurred at the time of binding. The ultrafiltration method allows the fixing of free and bound ligands [38]. So, the ratio of molar binding is calculated by Equation (1)

P = (K_m_ − K_n_)/R (1)

where P is the ratio of molar binding.

K_m_ is the total initial ligand.
K_n_ is the bound ligand.
R is the amount of total protein.

The ultrafiltration method is not suitable to analyze the interactions between the catechin dimers/trimers with proteins [67] because low solubility between them leads to the incorrect measurement of free (poly)phenol concentration. Therefore, it is revealed that the sensitivity of ultrafiltration varies with (poly)phenol structure. Understanding the interactions of (poly)phenols and milk proteins requires the usage of accurate and sophisticated methods.

### 7.2. Isothermal Titration Calorimetry

Isothermal titration calorimetry is a method that is mostly used for ligand binding. Most chemical reactions use changes in heat or enthalpy, and this method measures the heat change at a constant temperature and time of complex development, which is utilized in the titration method. ITC measurements are useful for the analysis of weak and strong ligands binding with the receptor, and it is also used for determining the characteristics of protein-(poly)phenol interactions [68]. Enthalpy–entropy compensation, which is a normal thermodynamic phenomenon indicated by enthalpy and entropy changes due to the negative correlation between some shared features, predicts binding affinity difficulty [69]. Moreover, the evaluation of the binding between bovine serum albumin with ferulic acid shows strong binding, and it was enthalpically and exothermically favored, which means the hydrogen bonds and electrostatic interactions play a vital role by using the model of two binding sites. The endothermic and hydrophobic interactions occur in the binding of catechins to β-casein, which shows a change in entropy over the enthalpy. The decrease in the hydrophobic sites on the surface of milk proteins after the addition of flavonoids indicates dominated hydrophobic interactions [38].

### 7.3. Molecular Docking

The active computational device for estimating the intermolecular complexes operates between two small molecules. This docking device explains suitable alignment to the molecules that form a stable compound and examines the strength of the binding reactions. The possible alignment for the binding of protein and small molecules at a minute point is generally referred to as “poses” [70]. Docking studies on proteins and ligands need the development of a complex model about the protein and ligand molecule structures. For the inference of protein structure, methods such as X-ray crystallography, NMR spectroscopy, and electron microscopy are used for the study of microscopic structures. Some studies review the molecular docking method for the predictable binding sites of (poly)phenols and milk proteins, and they reported useful devices for the binding characteristics between the (poly)phenols and the proteins found by other methods such as fluorescence quenching and isothermal titration calorimetry [69].

### 7.4. Thermodynamic Methods

Generally, four types of non-covalent interactions including hydrogen bonds, electrostatic interactions, hydrophobic interactions, and van der Waals forces occur between the macromolecules and tiny molecules. These forces of interaction can be determined by thermodynamic parameters (ΔS, ΔH, and ΔG), which indicate entropy change (ΔS), enthalpy changes (ΔH), and free energy change (ΔG), respectively. In this regard, the isothermal titration calorimetry can provide accurate information on existing binding sites when compared with other corresponding methods. Furthermore, the contribution of enthalpy and entropy to protein and ligand complexes, driving forces, and binding mechanisms in the interactions were also concluded. As such, ITC has unique features, and it is widely used for examining the binding reactions of several proteins and (poly)phenolic complexes [71]. For measuring the thermally induced properties of protein-(poly)phenolic complexes, differential scanning calorimetry (DSC) is widely used. During the interaction process, thermal stability brings changes in protein tertiary structures, and the stability of the complexes is either positively or negatively affected.

## 8. Biological Activity of (Poly)phenol-Milk Protein Complexes

The enhancement of milk products using plant-derived bioactive compounds has become a major interest of researchers and scientists over the past few years as a result of consumer demands for functional foods. They can be used in food processing for the development of physicochemical features, such as stability, texture, and the flavor of foods, or functional properties such as antioxidant or antimicrobial activity. The formulation of milk protein and (poly)phenolic complexes helps to increase the bioavailability and bioaccessibility of (poly)phenolic compounds and ultimately the food product as well. To avoid the degradation, loss, and unpleasant taste of (poly)phenols, the milk proteins are also used for an encapsulation technique. Some of the applications of this complex are antioxidant activity, anti-proliferative activity, and anti-carcinogenic properties, and these are discussed in detail.

### 8.1. Antioxidant Activity

Several techniques exist that reveal the antioxidant activity of the (poly)phenolic compound is due to the presence of the (poly)phenolic components consisting of three phenolic hydroxyl groups bound to a single benzene ring showing potential antioxidant properties. Furthermore, during gastrointestinal digestion, several factors such as enzyme activity, changes in pH, the nature of the compound, and interaction of dietary matrixes affect the bioavailability, bioactivity, and release of (poly)phenolic components. The interaction of (poly)phenols with protein complexes significantly improves the radical scavenging activity of (poly)phenolic compounds [72]. Besides, anthocyanins interact with the protein matrixes and remain protected during the time of transit through the upper alimentary canal, which allows the delivery of greater amounts to the large intestine. However, reports published by the researchers revealed both an increase and decline in the antioxidant activity of the (poly)phenolic compounds; however, it always depends on the type of proteins and (poly)phenolic compound. New key and important in vitro and in vivo findings revealed that milk proteins can be used as potential carriers for the plant-derived (poly)phenolic components and also protect them from oxidation reactions. Ultimately, this can increase the antioxidant activity and bioavailability of (poly)phenols in the gastrointestinal tract [73].

### 8.2. Anti-Proliferative Activity

Phenolic compounds with potential biological activities play a significant role in anticancer activity. Oxidative stress can cause DNA damage, therefore inducing mutations that might contribute to progressive cancer cell growth. Moreover, several reports also revealed the anti-proliferative effect of plant-derived (poly)phenolic compounds. Furthermore, several plant-based food components such as grapes, soybean, garlic, and olive effectively inhibit colorectal colon cancer cells [74]. Epigallocatechin gallate (EGCG) is a green tea (poly)phenolic extract, when added to milk, showed a potential reduction in the spread of colon cancer cells [75]. In addition, β-lactoglobulin is highly susceptible to substances of acidic nature and is easily breakdown by the gastric juices of the stomach. Herein, the β-lactoglobulin acts as a suitable milk protein component for the delivery of bioactive content, and due to this, an increase in the natural resistance of the whey protein from protein degradation occurs [43]. In many reports, it has been confirmed that the (poly)phenols can be carried to the inconsequential regions of the gastrointestinal tract, and due to that bioavailability and bio-accessibility increase significantly [76]. Undoubtedly, milk proteins and (poly)phenolic complexes can enhance the anti-proliferative activity of (poly)phenols.

### 8.3. Anti-Carcinogenic Properties

Some of the studies reported the anti-carcinogenic properties of (poly)phenols when interacting with the milk protein complexes. Overall, the addition of proteins affects the anti-carcinogenic properties of (poly)phenolic compounds in either positive, negative, or neutral ways [77]. Likewise, the interactions of some selected (poly)phenolic compounds and milk proteins reduce the cytotoxicity of tumor cells when compared to free (poly)phenols [78]. In particular, covalent bonding between the bovine serum albumin and sugarcane bagasse (poly)phenolic compounds reduces the probability of human colon cancer [79]. The formation of complexes with certain (poly)phenolic and milk protein molecules covalently may provide a novel drug delivery system towards the target cancer cells.

## 9. Impacts of (Poly)phenol and Protein Complexes on Food Quality

Commonly, most of the studies on protein-(poly)phenol complexes are focused on the physicochemical changes that occur during the interaction. In the presence of cocoa or coffee, (poly)phenolic compounds in milk exhibit few functional and technical properties of milk proteins such as thermal stability, foaming, solubility, gelation, and surface activity [38]. Before the fermentation of yogurt, the addition of blackcurrant (poly)phenolic compounds affects the colony number, morphology of the starter cultures, viscosity, and elasticity of set yogurts [79]. The interaction of (poly)phenols–protein complexes shows an impact on the sensory and color characteristics of many food systems. For example, the addition of milk in tea or coffee can decrease (poly)phenol accessibility [80]. However, milk proteins such as α-caseins and β-caseins show a positive effect on the photostability of grape seed extract (poly)phenols due to the non-covalent binding of (poly)phenols and milk protein complexes [81]. These above mentioned, significant findings are good examples of how the interactions of (poly)phenols and protein complexes impact the quality of products.

## 10. Conclusions and Future Perspective

Food is made up of several varieties of ingredients, and (poly)phenols and proteins are very important and abundantly present in food. A recent trend shows that people prefer new and attractive food ingredients with healthy food ingredients, which provide quality and nutraceutical value to the human body. Plant-derived (poly)phenols are secondary metabolites, and they are intensively investigated due to their potential effects on human health. However, environmental conditions and food processing limits the potential utilization of plant-derived (poly)phenolic compounds. Therefore, milk proteins are the most significant component in interactions with the (poly)phenolic compounds, and the interaction of (poly)phenols with milk significantly improves the bioactivity of the (poly)phenolic compounds. Moreover, the binding of (poly)phenolic compounds and milk proteins greatly influence the digestibility of proteins, the bioavailability of (poly)phenols, and essential amino acids. Besides, the positive aspects of milk protein (poly)phenolic complexes and commercialization of the food product are important. However, intensive research is still required for the development of staple food products carrying milk protein (poly)phenolic complexes. Food regulation bodies should also focus on the development of standards for the addition of vital complexes in food materials. Moreover, toxicological studies should also be conducted to obtain more information on the complexes.

## Figures and Tables

**Table 1:** Milk protein concentrates and their interactions with (poly)phenols.

Milk Protein Concentrates	(Poly)phenols	Type of Interaction	References
β-lactoglobulin	Tea (poly)phenols (catechin, epicatechin, epigallocatechin and epigallocatechin gallate)	Hydrophobic and Hydrophilic	[46]
Casein, whey proteins and β-lactoglobulin	Cocoa (poly)phenols (catechin and epicatechin)	Non-covalent bonding	[47]
Casien micelles and whey proteins	Black tea and green the (poly)phenols (catechin)	Hydrophobic	[48]
β-casein, α-casein, κ-casein, and whey protein	Coffee (poly)phenols (tannins)	Hydrogen bonding	[49]
α-caseins and β-caseins	Antioxidant (poly)phenols (resveratrol, genistein, and curcumin)	Hydrophilic and Hydrophobic	[50]
β-casein	Green tea (poly)phenols (catechin)	Hydrophobic, and non-covalent bonding	[43]
Casein and whey proteins	Green tea, grapes, and cranberry (poly)phenols (catechin, tannic acid, homovanillic acid, and hesperetin)	Hydrophobic	[51]

## Data Availability

Data sharing is not applicable to this article.
